# Barley Root Proteome and Metabolome in Response to Cytokinin and Abiotic Stimuli

**DOI:** 10.3389/fpls.2020.590337

**Published:** 2020-10-28

**Authors:** Miroslav Berka, Markéta Luklová, Hana Dufková, Veronika Berková, Jan Novák, Iñigo Saiz-Fernández, Aaron M. Rashotte, Břetislav Brzobohatý, Martin Černý

**Affiliations:** ^1^Department of Molecular Biology and Radiobiology, Faculty of AgriSciences, Mendel University in Brno, Brno, Czechia; ^2^Department of Biological Sciences, Auburn University, Auburn, AL, United States; ^3^Central European Institute of Technology, Faculty of AgriSciences, Mendel University in Brno, Brno, Czechia

**Keywords:** *Hordeum vulgare*, zeatin, proteome, metabolome, abiotic stress, phenylpropanoid biosynthesis, root, ROS

## Abstract

Cytokinin is a phytohormone involved in the regulation of diverse developmental and physiological processes in plants. Its potential in biotechnology and for development of higher-yield and more resilient plants has been recognized, yet the molecular mechanisms behind its action are far from understood. In this report, the roots of barley seedlings were explored as a new source to reveal as yet unknown cytokinin-responsive proteins for crop improvement. Here we found significant differences reproducibly observed for 178 proteins, for which some of the revealed cytokinin-responsive pathways were confirmed in metabolome analysis, including alterations phenylpropanoid pathway, amino acid biosynthesis and ROS metabolism. Bioinformatics analysis indicated a significant overlap between cytokinin response and response to abiotic stress. This was confirmed by comparing proteome and metabolome profiles in response to drought, salinity or a period of temperature stress. The results illustrate complex abiotic stress response in the early development of model crop plant and confirm an extensive crosstalk between plant hormone cytokinin and response to temperature stimuli, water availability or salinity stress.

## Introduction

Cytokinin is a multifaceted plant hormone that plays major roles in plant growth and development as well as interacting with both biotic and abiotic factors, including light, temperature, drought, and salinity signals (as reviewed in Argueso et al., 2009; Kieber and Schaller, 2014; Zwack and Rashotte, 2015; Pavlů et al., 2018; Cortleven et al., 2019). The canonical cytokinin signal transduction pathway is a multistep phosphorelay with sensor hybrid histidine kinase that phosphorylates histidine-containing phosphotransfer proteins which are then translocated into the nucleus, where they transfer the phosphate to type-B response regulators. The previous proteome-wide analyses have also revealed a transcription-independent rapid response to cytokinin targeting predominantly plastidial proteins (Chen et al., 2010; Černý et al., 2011, 2014; Žd’árská et al., 2013; Hallmark et al., 2020).

Seed germination and early seedling establishment is a complex biological process that includes drastic morphological, physiological, and biochemical changes, and represents a period of life when a plant is highly susceptible to both biotic and abiotic stressors (Bewley et al., 2013; Kaur et al., 2017). The early germination phase is thought of as predominantly governed by the phytohormones abscisic acid and gibberellin, yet other growth regulators are also known to participate in the complex process. The role of cytokinin in seedling establishment is indisputable, including regulation of shoot and root apical meristems, development of lateral roots or vasculature system, light signaling, chloroplast development and de-etiolation and leaf development (Chory et al., 1994; Kieber and Schaller, 2014; Skalák et al., 2019). Early reports of Arabidopsis cytokinin mutants showed that a receptor mutation or an enhanced cytokinin degradation resulted in more rapid germination (Riefler et al., 2006). In contrast, later studies revealed that cytokinin antagonizes abscisic acid by downregulating ABI5 and promoting its degradation (Wang et al., 2011; Guan et al., 2014) and found that plants overexpressing cytokinin response regulators type-A were insensitive to abscisic acid (Huang et al., 2017).

Barley refers to the cereal *Hordeum vulgare* subsp. *vulgare* and belongs to the Triticeae tribe of grasses. The tribe comprises about 350 species among them the important cereals are wheat (*Triticum* spp.), rye (*Secale cereale*) and triticale (x*Triticosecale*), many forage grasses and ecologically important taxa of temperate grasslands (Blattner, 2018). Barley is the fourth most important cereal grain with a worldwide production exceeding 141 million tonnes (FAOSTAT, year 2018; http://faostat.fao.org) and an excellent crop model species with a sequenced genome (Mayer et al., 2012). Barley has a diploid genome (2n) with only 14 chromosomes making it more suitable for analyses than its close relative from Triticeae tribe, wheat. There are over 400,000 barley accessions conserved in collections world-wide (Dawson et al., 2015) and biotechnology tools for transformation have been developed (e.g., Harwood, 2019). Previous research on cytokinin signaling has successfully developed transgenic barley lines with an enlarged root system, higher yield and improved drought resilience (Zalewski et al., 2010; Mrízová et al., 2013; Pospíšilová et al., 2016; Vojta et al., 2016; Holubová et al., 2018; Ramireddy et al., 2018), but analyses targeting molecular mechanisms underlying cytokinin response in barley are limited. The Arabidopsis Information Resource lists 142 genes associated with cytokinin. In contrast, only 20 and 30 genes are associated with the cytokinin signaling or metabolism in the reference barley genomes IBSC and Morex, respectively (plants.ensembl.org; barlex.barleysequence.org).

Cytokinin is one of the principal players in the process of root organogenesis (e.g., Laplaze et al., 2007; Müller and Sheen, 2008; Bielach et al., 2012; Kurepa et al., 2019) and roots are thus an excellent target for the cytokinin response analysis. Further, the detection of plant proteins is often hindered by the presence of Ribulose bisphosphate carboxylase/oxygenase (RubisCO) which may represent more than 50% of the plant protein extracts (e.g., Righetti and Boschetti, 2016). Techniques have been developed to eliminate or deplete this highly abundant enzyme (Gupta et al., 2015) but the root tissue, being devoid of RubisCO, does not require this additional step. We hypothesized that barley seedling root could be an excellent model for analysis of cytokinin-responsive proteins and that comprehensive, integrated and high-resolution analyses could extend current understanding of cytokinin role in early plant development. To elicit the cytokinin response, we selected the treatment with trans-zeatin, which is together with cis-zeatin the dominant active cytokinin in barley (Powell et al., 2013).

## Results

### Barley Root Proteome

**FIGURE 1:**
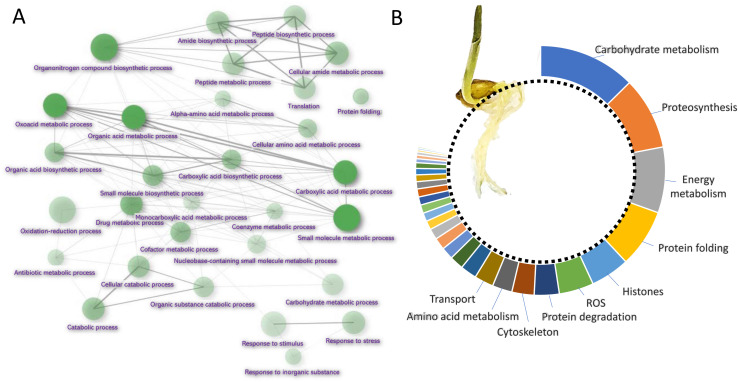
Barley root proteome composition. **(A)** Enrichment analysis based on hypergeometric distribution followed by FDR correction. Two pathways are connected if they share 20% or more proteins. Darker and bigger nodes are more significantly enriched and larger sets, respectively. Analyzed by ShinyGO 0.61 (Ge et al., 2020); **(B)** The function of the most abundant proteins representing 80% of the barley root proteome. For the sake of clarity, only ten most abundant categories are labeled.

Barley root proteome was extracted from roots of four-day-old seedlings as described in Materials and Method. Root proteome profiling identified over 10,000 peptides corresponding to more than 2,600 proteins represented by 1,735 protein families. The gene ontology (GO) analysis revealed that most proteins are identified as being involved in biosynthetic processes, including amino acid biosynthesis, proteosynthesis and the production of secondary metabolites (Figures 1A,B). Proportionally, the most abundant proteins representing 80% of the analyzed root proteome comprised of only 460 proteins and the majority of these was formed by carbohydrate metabolism enzymes (15.2%), ribosomal proteins and proteins involved in proteosynthesis (11.3%), energy metabolism (10.3%), protein folding (9.1%), histones (6.1%) and reactive oxygen species (ROS) metabolism (5.4%). There was also a noticeable amount of jasmonic acid (JA)-responsive proteins and JA biosynthetic enzymes (12-oxophytodienoate reductase, multiple lipoxygenases), representing more than 2.3% of all detected proteins.

### Cytokinin at Micromolar Range Does Not Have a Negative Impact on Barley Germination

**FIGURE 2:**
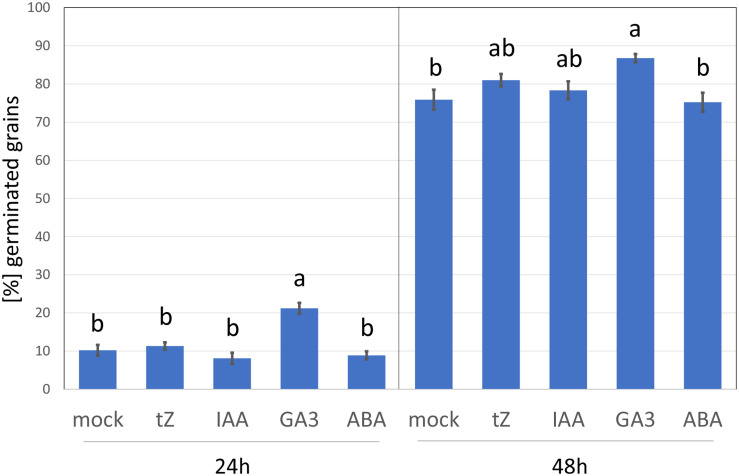
Barley germination in response to different growth regulators. Percentage of germinated seeds 24 and 48 h after imbibition in 4 ml water supplemented with 5 × 10^–4^% (v/v) dimethyl sulfoxide (mock buffer) or 1 μM phytohormone (tZ, trans-Zeatin; IAA, Indole-3-acetic acid; GA3, Gibberellic acid; ABA, abscisic acid) in dimethyl sulfoxide (final concentration, as for the mock). Different letters indicate significant differences (Kruskal-Wallis test, *p* < 0.05).

To elicit the cytokinin response, barley seedlings were treated with 1 μM of the cytokinin, trans-zeatin for 24 h. This is a similar concentration to that used in previous cytokinin transcriptomics and proteomics analyses but far above the natural endogenous levels (Černý et al., 2016). The addition of higher cytokinin concentrations may induce a hypersensitive-like response and promote cell death (Vescovi et al., 2012; Novák et al., 2013), and thus it cannot be excluded that some of the observed cytokinin-responsive proteins found here could represent a response to toxicity. However, as illustrated in Figure 2, the barley germination rate in the presence of 1 μM trans-zeatin was similar to that of mock-treated grains, and the percentage of germinated grains after 48 h was unaffected. This standard assay evaluates only the emergence of the radicle, but it is likely that barley seedlings can counter any negative effect of cytokinin at this concentration and duration by triggering its degradation, which is supported by the fact that cytokinin-treated roots accumulated a sufficient amount of cytokinin dehydrogenase (CKX6) to facilitate its detection in an untargeted proteome-wide analysis (Supplementary Table S2).

### Cytokinin-Responsive Proteins in Barley Root Proteome

**FIGURE 3:**
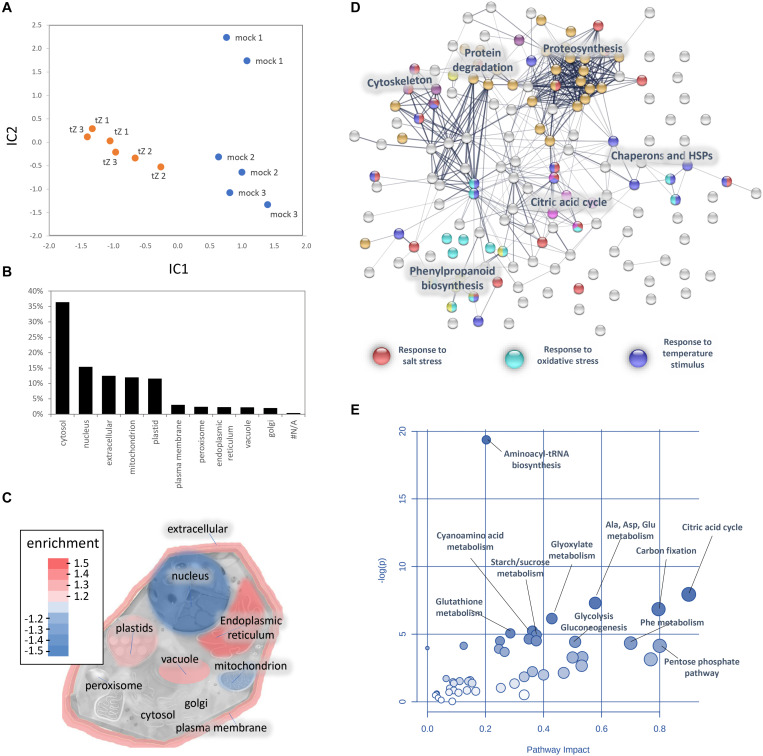
Cytokinin impact on the barley root proteome and metabolome. **(A)** The proteome profile separation of cytokinin- and mock-treated samples. Independent component analysis based on quantitative data of 972 proteins. Results of three biological replicates represent proteins with at least 10 matched spectra in a biological replicate and more than > 95% of the detected root proteome. **(B)** Expected localization of quantified barley root proteins and **(C)** visualization of organellar enrichment in cytokinin-responsive proteins. Localization based on data prediction by CropPal and SUBA4.0 (Hooper et al., 2016, 2017); **(D)** Interactions and functional clusters of cytokinin-responsive proteins highlighted by String (Szklarczyk et al., 2019). The selected highlighted categories are represented by at least five and ten proteins for ‘Biological Processes’ and ‘KEGG Pathways,’ respectively; **(E)** Cytokinin impact on barley root metabolism. Highlighted pathways were identified by an integrative analysis of identified cytokinin-responsive proteins and metabolites (MetaboAnalyst; Pang et al., 2020). Results (D-E) are based on data available for putative Arabidopsis orthologs.

**FIGURE 4:**
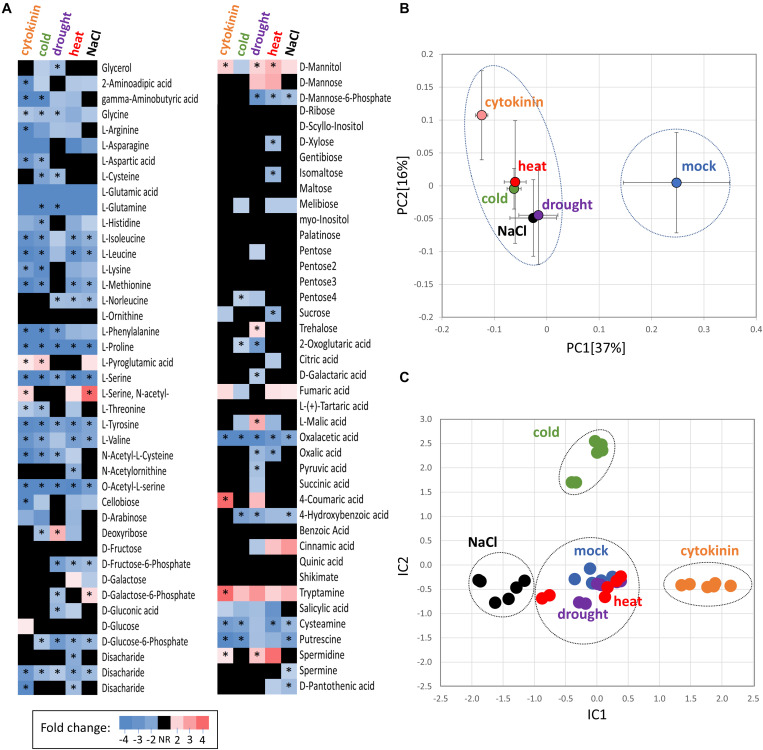
Comparison of cytokinin- and abiotic stress-induced changes in roots of barley. **(A)** Heat map visualization of detected root metabolites. Asterisks indicate statistically significant changes (*p* < 0.05); **(B)** Similarities in the average metabolome composition visualized by PCA. Presented data are means and standard error (*n* = 6); **(C)** Clusters of treatments obtained from independent component analysis of protein profiles; Dashed circles represent statistically significant separation (Kruskal-Wallis test, *p* < 0.05).

A comparison of proteome profiles between cytokinin and mock-treated roots showed significant separation (Figure 4A) and revealed 350 candidate cytokinin-responsive proteins (p < 0.05), including the above-mentioned enzyme CKX6 (found only in cytokinin-treated roots). Next, candidates failing stringent identification and quantitation criteria were removed and only differentially abundant proteins with at least 10 spectral matches in a biological replicate, more than one unique peptide and absolute fold-change of at least 1.4 were selected, limiting the set to 178 differentially abundant cytokinin-responsive proteins (Tables 1, 2). In total, 63 and 115 cytokinin-responsive proteins representing more than 12% of the total root proteome were found as significantly increased and decreased, respectively. The most numerous categories also matched the total proteome composition (carbohydrate metabolism, ribosomal proteins and proteosynthesis, energy metabolism; Figure 3D). Predicted cellular localization showed that the cytokinin-responsive proteins were found predominantly in the cytosol (35%), extracellular space (16%), plastids (14%), nucleus (10%), and mitochondrion (9%). Comparisons of cytokinin-responsive localization to the whole proteome dataset showed that mitochondrial and nuclear proteins were underrepresented (Figures 3B,C). The set of cytokinin-induced proteins included enzymes of phenylpropanoid biosynthesis, components of cytoskeleton and enzymes of lipid metabolism. The metabolic pathway enrichment revealed that proteins with a significant depletion were enriched in ribosomal proteins (11), enzymes of biosynthesis of secondary metabolites (17) or protein processing (8). About one-third of the cytokinin-repressed proteins was associated with a stress response, indicating a putative connection between cytokinin-induced alleviation or attenuation of the stress. Not all identified differentially abundant proteins are novel cytokinin-responsive proteins *per se*. The comparison to previously known cytokinin-responsive proteins identified at least 26 barley root proteins orthologs that were found with a similar response to cytokinin (Lochmanová et al., 2008; Liu et al., 2011; Černý et al., 2011, 2014; Zhang et al., 2012; Žd’árská et al., 2013; Hallmark et al., 2020) and 36 cytokinin-responsive genes from *Arabidopsis thaliana* (Hoth et al., 2003; Brenner et al., 2005; Kiba et al., 2005; Nemhauser et al., 2006; Argyros et al., 2008; Li et al., 2010; Brenner and Schmulling, 2012; Nishiyama et al., 2012; Bhargava et al., 2013).

**TABLE 1:** Proteins with a positive response to cytokinin.

	Accession	Name	AGI	CK	tZ	D	C	H	S
Amino acid metabolism	HORVU5Hr1G104700	Glutamate dehydrogenase 1	N/A		↑	NR	NR	NR	NR
	HORVU2Hr1G029870	Phospho-2-dehydro-3-deoxyheptonate aldolase 2	AT1G22410		↑	↑	NR	NR	↑
Biosynthesis of secondary metabolites	HORVU3Hr1G015640	Alkenal reductase	AT5G16990		↑	NR	NR	NR	NR
	HORVU6Hr1G035420	Cinnamyl alcohol dehydrogenase 5	AT3G19450		↑	NR	NR	NR	↑
	HORVU7Hr1G082280	O-Methyltransferase 1	AT5G54160	×	↑	NR	NR	NR	NR
	HORVU6Hr1G058820	Phenylalanine ammonia-lyase 2	AT2G37040	×	↑	NR	NR	↑	↑
	HORVU0Hr1G016330	Phenylalanine ammonia-lyase 2	AT2G37040	×	↑	NR	NR	↑	↑
	HORVU2Hr1G117770	Sulfotransferase 2A	AT5G07010	×	↑	NR	↓	NR	NR
Carbohydrate metabolism	HORVU7Hr1G001040	Acid β-fructofuranosidase	AT1G12240		↑	↑	NR	NR	↓
	HORVU6Hr1G074200	Dihydroorotate dehydrogenase (quinone)	AT3G17810		↑	NR	NR	NR	NR
	HORVU3Hr1G002780	Fructose-bisphosphate aldolase 2	AT2G21330	×	↑	NR	NR	↑	NR
	HORVU7Hr1G013600	Transketolase	AT2G45290		↑	NR	NR	↑	NR
Cell cycle	HORVU4Hr1G072010	Cell division cycle 48	AT5G03340		↑	NR	NR	↑	NR
Cell wall	HORVU1Hr1G054240	Expansin B2	AT1G65680		↑	↑	NR	NR	NR
Cytoskeleton	HORVU1Hr1G081280	Tubulin α-4 chain	AT4G14960	×	↑	NR	NR	NR	NR
	HORVU3Hr1G078940	Tubulin β chain 2	AT5G12250	×	↑	NR	NR	NR	NR
	HORVU4Hr1G002530	Tubulin β chain 4	AT2G29550	×	↑	NR	NR	NR	NR
	HORVU5Hr1G100900	Tubulin β chain 5	AT1G20010	×	↑	NR	NR	NR	NR
Energy metabolism	HORVU6Hr1G088420	2-Oxoglutarate (2OG) and Fe(II)-dependent oxygenase	AT1G49390		↑	NR	NR	NR	NR
	HORVU1Hr1G083470	5-Dehydro-2-deoxygluconokinase	AT4G10260		↑	NR	NR	NR	NR
	HORVU1Hr1G090720	Malate dehydrogenase	AT1G53240	×	↑	NR	↑	NR	NR
	HORVU5Hr1G111620	Methylenetetrahydrofolate reductase 2	AT2G44160		↑	↑	NR	NR	NR
Histone	HORVU1Hr1G074340	Histone superfamily protein	AT1G09200		↑	NR	NR	NR	NR
	HORVU6Hr1G031580	Histone superfamily protein	AT1G09200		↑	NR	NR	NR	NR
Chaperon	HORVU2Hr1G092190	Hsp70-Hsp90 organizing protein	AT1G62740		↑	↑	↑	NR	NR
Lipid metabolism	HORVU7Hr1G119060	GDSL esterase/lipase	AT3G04290	×	↑	NR	NR	NR	NR
	HORVU6Hr1G074940	Lipase/lipooxygenase	AT4G39730		↑	NR	NR	NR	NR
	HORVU1Hr1G012140	PATATIN-like protein 4	AT2G26560	×	↑	NR	NR	NR	NR
	HORVU7Hr1G036460	PATATIN-like protein 4	AT2G26560	×	↑	NR	NR	NR	NR
Nucleic acid and nucleotide metabolism	HORVU3Hr1G077000	Adenine nucleotide α hydrolase	AT5G54430		↑	NR	NR	NR	NR
	HORVU6Hr1G079150	Adenine nucleotide α hydrolase	AT3G03270	×	↑	NR	↑	NR	NR
	HORVU5Hr1G109340	ATP-dependent RNA helicase DED1	AT2G42520		↑	NR	NR	↑	NR
	HORVU2Hr1G003110	Ectonucleoside triphosphate diphosphohydrolase 5	AT3G04080	×	↑	NR	↓	NR	NR
	HORVU4Hr1G087230	Ectonucleoside triphosphate diphosphohydrolase 5	AT3G04080		↑	NR	↓	↓	↓
	HORVU2Hr1G112830	Nuclease S1	AT1G68290		↑	NR	↓	NR	↓
Phosphatase	HORVU4Hr1G087310	Acid phosphatase	N/A		↑	NR	NR	NR	NR
	HORVU4Hr1G085050	Purple acid phosphatase 27	AT5G50400		↑	NR	↓	NR	↓
Protease	HORVU7Hr1G009410	Eukaryotic aspartyl protease	AT1G01300	×	↑	NR	NR	NR	NR
	HORVU6Hr1G081640	Subtilisin-like protease	AT3G14067		↑	NR	NR	NR	↓
ROS	HORVU6Hr1G008640	Catalase 1	AT1G20620		↑	NR	NR	NR	NR
	HORVU7Hr1G121700	Catalase 2	AT4G35090	×	↑	NR	NR	NR	NR
	HORVU4Hr1G081100	Glutathione S-transferase family protein	AT3G03190	×	↑	NR	NR	NR	NR
	HORVU1Hr1G016660	Peroxidase superfamily protein	AT4G11290		↑	↓	↓	↓	↓
	HORVU2Hr1G124970	Peroxidase superfamily protein	AT1G71695	×	↑	NR	NR	↓	↓
Signaling	HORVU2Hr1G077220	12-Oxophytodienoate reductase 2	AT1G09400		↑	NR	NR	NR	↑
	HORVU4Hr1G005920	Lipoxygenase 1	AT1G55020	×	↑	NR	↓	NR	NR
	HORVU5Hr1G093770	Lipoxygenase 1	AT1G17420	×	↑	NR	NR	NR	NR
Stress response	HORVU6Hr1G083960	Dehydrin	AT3G50970	×	↑	NR	NR	NR	NR
	HORVU5Hr1G010880	Germin-like protein 2	AT5G38910	×	↑	NR	NR	NR	NR
	HORVU0Hr1G011720	Major pollen allergen bet v 1-B	AT1G35260		↑	NR	NR	NR	NR
	HORVU5Hr1G023720	Pathogenesis-related protein STH-2	AT1G35260		↑	↑	NR	NR	NR
	HORVU2Hr1G120530	Wound-induced protein	AT3G04720		↑	NR	NR	NR	NR
	HORVU3Hr1G113620	Wound-induced protein	AT3G04720		↑	NR	NR	NR	NR
Transport	HORVU6Hr1G074440	Annexin 7	AT5G10230		↑	↑	↓	↑	↑
	HORVU6Hr1G091250	Voltage-gated potassium channel subunit β-1	AT1G04690	×	↑	↑	NR	NR	↑
Unknown	HORVU7Hr1G021430	HXXXD-type acyl-transferase family protein	AT1G31490	×	↑	↑	↓	NR	↓
	HORVU7Hr1G039930	NAD(P)-linked oxidoreductase superfamily protein	AT1G60690		↑	NR	NR	NR	NR
	HORVU1Hr1G043910	Plant basic secretory protein	AT2G15130		↑	NR	NR	NR	NR
	HORVU1Hr1G043920	Plant basic secretory protein	AT2G15220	×	↑	NR	NR	NR	NR
	HORVU6Hr1G013710	Plant basic secretory protein	AT2G15220	×	↑	NR	NR	NR	↓
	HORVU2Hr1G081080	UBX domain-containing protein	AT4G04210		↑	NR	NR	NR	NR
	HORVU5Hr1G073650	Unknown	AT1G09310		↑	NR	NR	NR	NR
	HORVU5Hr1G024040	Zinc-binding dehydrogenase family protein	AT3G03080		↑	↑	NR	NR	NR

*AGI, Arabidopsis best match; CK – (×) found with a similar response to cytokinin in previous analyses; Arrows indicate protein accumulation (↑) or depletion (↓) in response to cytokinin (tZ), drought (D), period of cold (C) or heat (H) stress or salinity (S). See Supplementary Tables S1, S2 for details.*

**TABLE 2:** Proteins with a negative response to cytokinin.

	Accession	Name	AGI	CK	tZ	D	C	H	S
Amino acid metabolism	HORVU7Hr1G051070	Aminoacylase-1	AT4G38220		↓	NR	↓	↓	NR
	HORVU4Hr1G056240	Asparagine synthetase [glutamine-hydrolyzing] 1	AT3G47340		↓	↑	NR	NR	↑
	HORVU3Hr1G073220	Aspartate aminotransferase 3	AT5G11520	×	↓	NR	NR	NR	NR
	HORVU5Hr1G048890	Carbamoyl-phosphate synthase small chain	AT3G27740		↓	↓	NR	NR	NR
	HORVU4Hr1G016410	Methylthioribose-1-phosphate isomerase	AT2G05830		↓	NR	NR	NR	NR
Biosynthesis of secondary metabolites	HORVU2Hr1G086080	6,7-Dimethyl-8-ribityllumazine synthase	AT2G44050		↓	↓	↓	NR	NR
	HORVU5Hr1G100910	Inosine-5′-monophosphate dehydrogenase	AT1G16350		↓	NR	NR	↓	NR
	HORVU3Hr1G032400	D-Alanine aminotransferase	AT5G57850		↓	↓	↓	NR	↓
Carbohydrate metabolism	HORVU1Hr1G080480	6-Phosphogluconate dehydrogenase, decarboxylating 1	AT1G64190		↓	↓	↓	NR	NR
	HORVU7Hr1G000250	Acid β-fructofuranosidase	AT1G12240		↓	NR	↓	NR	↑
	HORVU1Hr1G070310	Aldose reductase	AT2G37760		↓	↓	NR	↓	NR
	HORVU1Hr1G088560	GDP-D-mannose 3′,5′-epimerase	AT5G28840	×	↓	NR	NR	NR	NR
	HORVU5Hr1G069850	Glucose-6-phosphate isomerase	AT4G24620		↓	↓	↓	NR	↓
	HORVU3Hr1G073780	α-1,4-Glucan-protein synthase [UDP-forming]	AT3G02230	×	↓	NR	NR	NR	NR
	HORVU6Hr1G078330	α-Amylase	AT4G25000		↓	NR	↓	NR	NR
	HORVU6Hr1G080790	α-Amylase	AT4G25000		↓	NR	↓	NR	NR
	HORVU5Hr1G113880	α-Mannosidase	AT3G26720		↓	NR	NR	↓	↓
	HORVU1Hr1G057680	β-1,3-Glucanase	AT3G57260	×	↓	NR	NR	↓	NR
	HORVU6Hr1G075010	β-D-xylosidase 4	AT1G78060	×	↓	↓	NR	NR	↓
	HORVU5Hr1G095080	β-Glucosidase C	AT5G20950	×	↓	↓	NR	NR	NR
Cell cycle	HORVU4Hr1G033200	Translationally controlled tumor protein	AT3G16640		↓	NR	NR	NR	NR
Cytoskeleton	HORVU5Hr1G113060	Actin depolymerizing factor 4	AT1G01750	×	↓	NR	NR	NR	NR
	HORVU4Hr1G047580	H/ACA ribonucleoprotein complex subunit 1-like protein 2	AT5G07760		↓	NR	NR	NR	NR
	HORVU7Hr1G052350	Myosin heavy chain	AT4G31340	×	↓	NR	↓	NR	NR
Energy metabolism	HORVU5Hr1G078540	2Fe-2S Ferredoxin-like superfamily protein	AT3G07480		↓	↓	NR	↓	↓
	HORVU2Hr1G070090	2-Oxoglutarate dehydrogenase	AT5G65750		↓	NR	↓	NR	NR
	HORVU4Hr1G080640	Aconitate hydratase 1	AT2G05710	×	↓	NR	↓	NR	NR
	HORVU4Hr1G016810	Alcohol dehydrogenase 1	AT1G64710	×	↓	NR	NR	↑	NR
	HORVU5Hr1G112850	Dihydrolipoyllysine-residue acetyltransferase component of pyruvate dehydrogenase complex	AT3G25860		↓	NR	↓	NR	NR
	HORVU2Hr1G006250	Dihydrolipoyllysine-residue succinyltransferase	N/A		↓	NR	NR	NR	NR
	HORVU2Hr1G103180	L-Lactate dehydrogenase	N/A		↓	NR	↓	NR	↓
	HORVU1Hr1G066240	NAD(P)H dehydrogenase (quinone)	AT5G54500		↓	↓	NR	NR	NR
	HORVU3Hr1G077250	NAD(P)H dehydrogenase (quinone)	AT5G54500		↓	↓	↓	↓	↓
	HORVU0Hr1G013850	Succinate dehydrogenase	AT5G66760		↓	NR	NR	NR	NR
Histone	HORVU5Hr1G082190	Histone H2A 6	AT1G51060	×	↓	NR	NR	NR	NR
LEA protein	HORVU4Hr1G051780	LEA protein 1	AT2G36640	×	↓	NR	NR	↓	NR
	HORVU3Hr1G039050	LEA protein-like	AT2G36640	×	↓	NR	NR	NR	NR
Nitrogen metabolism	HORVU6Hr1G080750	Ferredoxin–nitrite reductase	AT2G15620		↓	↓	↓	NR	NR
Nucleic acid and nucleotide metabolism	HORVU2Hr1G085880	Lupus La protein homolog A	AT4G32720		↓	NR	↓	NR	NR
	HORVU4Hr1G016440	Multiple organellar RNA editing factor 1	AT4G20020		↓	↓	NR	NR	NR
	HORVU7Hr1G063970	Polyadenylate-binding protein 2	AT4G34110		↓	NR	↓	NR	NR
	HORVU5Hr1G110370	RNA-binding protein 1	AT5G61030	×	↓	↓	↓	NR	↓
	HORVU6Hr1G091860	rRNA/tRNA 2’-O-methyltransferase fibrillarin-like protein 1	AT5G52470		↓	NR	NR	NR	NR
	HORVU4Hr1G005100	Tudor domain-containing protein 1	AT5G07350		↓	NR	↓	NR	NR
Protein degradation	HORVU4Hr1G010300	Cathepsin B-like cysteine proteinase 5	AT1G02300		↓	NR	NR	NR	NR
	HORVU5Hr1G076130	Proteasome subunit β type-1	AT3G60820		↓	NR	NR	NR	NR
	HORVU0Hr1G040340	Proteasome subunit β type-2	AT4G14800		↓	NR	NR	NR	NR
	HORVU5Hr1G078810	Proteasome subunit β type-4	AT1G56450	×	↓	NR	NR	NR	NR
	HORVU5Hr1G065170	Related to ubiquitin 1	AT1G31340		↓	NR	NR	NR	NR
	HORVU1Hr1G089380	Subtilisin-like protease	AT1G32960		↓	NR	↓	↓	↓
	HORVU1Hr1G023660	Ubiquitin 5	AT1G23410		↓	NR	NR	NR	NR
Protein folding and chaperons	HORVU5Hr1G062310	10 kDa chaperonin	AT5G20720		↓	NR	NR	↓	NR
	HORVU5Hr1G125130	60 kDa chaperonin 2	AT5G18820		↓	NR	NR	NR	NR
	HORVU7Hr1G082540	Copper chaperone	AT1G66240		↓	NR	NR	NR	NR
	HORVU1Hr1G087070	DnaJ homolog subfamily B member 13	AT2G20560		↓	↓	↑	NR	NR
	HORVU5Hr1G078400	HSP 70 C	AT5G42020	×	↓	NR	NR	NR	NR
	HORVU4Hr1G012460	Chaperone protein DnaK	AT4G24280		↓	NR	NR	NR	NR
	HORVU4Hr1G089090	Chaperone protein DnaK	AT4G24280		↓	NR	NR	NR	NR
	HORVU5Hr1G075490	Peptidyl-prolyl cis-trans isomerase	AT3G25220	×	↓	↓	NR	↓	NR
	HORVU6Hr1G077340	Peptidyl-prolyl cis-trans isomerase	AT5G64350	×	↓	NR	↑	↓	NR
	HORVU3Hr1G020490	sHSP 17.6 kDa class II	AT5G12020		↓	NR	↑	NR	NR
	HORVU3Hr1G020500	sHSP 17.6 kDa class II	AT5G12020		↓	NR	↑	NR	NR
	HORVU3Hr1G020520	sHSP 17.6 kDa class II	AT1G54050		↓	NR	↑	NR	NR
	HORVU4Hr1G063350	sHSP 21	AT4G27670		↓	↓	↑	↓	NR
Protein inhibitor	HORVU5Hr1G111920	Serpin-Z7	AT1G47710		↓	NR	NR	NR	NR
Peptide metabolism	HORVU6Hr1G084960	Leucine aminopeptidase 1	N/A		↓	NR	↓	NR	↓
Proteosynthesis	HORVU4Hr1G023570	30S ribosomal protein S9	AT2G09990		↓	NR	NR	↑	NR
	HORVU1Hr1G042220	40S ribosomal protein S17-4	AT5G04800		↓	NR	NR	NR	↑
	HORVU3Hr1G115820	40S ribosomal protein S28	AT3G10090		↓	NR	↑	↓	NR
	HORVU4Hr1G070370	40S ribosomal protein S3a	AT4G34670		↓	NR	NR	NR	NR
	HORVU2Hr1G029890	40S ribosomal protein S6	AT5G10360	×	↓	NR	NR	NR	NR
	HORVU4Hr1G055230	40S ribosomal protein S7	AT3G02560		↓	NR	NR	NR	NR
	HORVU1Hr1G028820	50S ribosomal protein L5	AT2G42740		↓	NR	NR	NR	NR
	HORVU7Hr1G063280	60S ribosomal protein L27-3	AT4G15000		↓	NR	NR	NR	↑
	HORVU3Hr1G038950	60S ribosomal protein L30	N/A		↓	NR	NR	NR	NR
	HORVU7Hr1G031850	60S ribosomal protein L35a-3	AT1G74270		↓	NR	NR	NR	NR
	HORVU1Hr1G021130	Asparagine–tRNA ligase	AT5G56680		↓	↑	↓	↑	NR
	HORVU7Hr1G080870	Elongation factor Ts	AT4G11120		↓	NR	NR	NR	NR
	HORVU5Hr1G122350	Elongation factor Tu	AT4G02930		↓	NR	NR	NR	NR
	HORVU4Hr1G050630	Eukaryotic translation initiation factor 2 β subunit	AT5G20920	×	↓	NR	NR	NR	NR
	HORVU6Hr1G012010	Eukaryotic translation initiation factor 3 subunit J-A	AT5G37475		↓	NR	NR	↓	↓
	HORVU1Hr1G057970	Nascent polypeptide-associated complex subunit α-like protein 3	AT3G12390		↓	↓	NR	↓	↓
	HORVU3Hr1G001140	Ribosomal protein L6 family	AT1G33120		↓	NR	NR	NR	NR
	HORVU5Hr1G034770	Ribosomal protein S3 family protein	AT5G35530		↓	↑	NR	↑	NR
ROS	HORVU5Hr1G103180	Ferredoxin–NADP reductase	AT1G30510	×	↓	↓	NR	↓	NR
	HORVU3Hr1G023970	Glutaredoxin 4	AT3G15660		↓	NR	NR	NR	NR
	HORVU6Hr1G080770	Lipid-binding protein	AT5G42890	×	↓	NR	NR	↓	NR
	HORVU2Hr1G083170	Peptide methionine sulfoxide reductase MsrA	AT5G07470		↓	NR	NR	↓	↑
	HORVU2Hr1G125200	Peroxidase superfamily protein	N/A		↓	↓	NR	NR	NR
	HORVU3Hr1G112350	Peroxidase superfamily protein	AT1G71695	×	↓	NR	NR	NR	NR
	HORVU7Hr1G010280	Peroxidase superfamily protein	AT1G05260	×	↓	NR	NR	↑	NR
	HORVU3Hr1G037720	Peroxiredoxin-2D	AT3G06050		↓	↓	↓	NR	NR
	HORVU7Hr1G046280	Thioredoxin domain-containing protein 17	AT5G42850		↓	↓	↓	↓	↓
Signaling	HORVU1Hr1G001850	12-oxophytodienoate reductase 2	AT1G17990		↓	NR	↓	↓	↑
	HORVU5Hr1G067710	Jasmonate-induced protein	AT3G21380		↓	↓	↓	NR	↓
	HORVU1Hr1G045630	Membrane steroid binding protein 1	AT5G52240		↓	NR	↓	NR	↓
Storage protein	HORVU1Hr1G008130	11S seed storage protein	AT2G28680	×	↓	NR	↓	NR	NR
	HORVU5Hr1G104630	Vicilin	AT3G22640		↓	NR	NR	NR	NR
	HORVU4Hr1G070970	Vicilin-like antimicrobial peptides 2-2	AT2G18540		↓	NR	NR	NR	NR
Stress response	HORVU6Hr1G089570	Germin-like protein 5	AT1G02335		↓	NR	NR	NR	↓
	HORVU6Hr1G089510	Germin-like protein 5	AT1G02335		↓	NR	↓	NR	↓
	HORVU4Hr1G054920	LL-Diaminopimelate aminotransferase	AT2G13810		↓	NR	NR	NR	NR
Transport	HORVU6Hr1G070780	ADP,ATP carrier protein, mitochondrial	AT5G13490	×	↓	NR	↓	↓	↓
	HORVU3Hr1G009370	Non-specific lipid-transfer protein 4.1	AT5G59310		↓	NR	↓	↓	NR
	HORVU7Hr1G054440	Nuclear transport factor 2A	AT1G27970		↓	NR	↓	NR	NR
	HORVU2Hr1G109500	Protein transport protein SEC13 homolog A	AT2G30050		↓	NR	↓	NR	NR
Unknown	HORVU5Hr1G042310	ACT domain-containing protein	AT5G04740	×	↓	NR	NR	NR	NR
	HORVU1Hr1G000940	Copper ion binding protein	AT4G12340		↓	↓	NR	↓	NR
	HORVU1Hr1G080980	Hyaluronan/mRNA binding family	AT4G17520		↓	NR	↑	NR	NR
	HORVU5Hr1G073450	NAD(P)-binding Rossmann-fold superfamily protein	AT5G10730	×	↓	NR	NR	↓	NR
	HORVU7Hr1G003710	RNA-binding (RRM/RBD/RNP motifs) family protein	AT5G16840		↓	NR	NR	NR	NR
	HORVU2Hr1G056740	Small nuclear ribonucleoprotein associated protein B	AT4G20440		↓	NR	NR	NR	NR
	HORVU2Hr1G098860	Unknown	AT2G32240	×	↓	↓	↓	↓	↓
	HORVU7Hr1G080350	Unknown	N/A		↓	NR	NR	NR	NR
	HORVU1Hr1G082820	Unknown	AT3G53040		↓	NR	NR	↓	NR

*AGI, Arabidopsis best match; CK, (×) found with a similar response to cytokinin in previous analyses; Arrows indicate protein accumulation (↑) or depletion (↓) in response to cytokinin (tZ), drought (D), period of cold (C) or heat (H) stress or salinity (S). See Supplementary Tables S1, S2 for details.*

### Cytokinin-Responsive Metabolites in Roots of Barley Seedling

A GC-MS metabolome analysis for barley seedling roots provided quantitative data for 80 different root metabolites, giving a snapshot of the key primary metabolism pathways as well as phenylpropanoid biosynthesis or polyamine production (Figure 4A, Supplementary Table S3). The cytokinin treatment induced a significant accumulation of N-acetyl serine, mannitol, pyroglutamic acid, coumaric acid, polyamine spermidine, and tryptamine. The accumulation of an osmoprotective compound (mannitol), the product of glutathione degradation (pyroglutamic acid), phenolic compound (coumaric acid), and an important precursor for many secondary metabolites (tryptamine) was matching the observed accumulation of stress-responsive proteins. In parallel, the set of 22 metabolites that were significantly depleted in response to cytokinin fully supported observed changes on the proteome level. Namely, the depletion of oxaloacetate and aspartate correlated with a decrease in aspartate aminotransferase and asparagine synthase. The depletion of aminoacylase and alanine aminotransferase coincided with a decrease in arginine and proline. And, finally, a decrease in lysine was likely the result of diaminopimelate aminotransferase repression. The observed accumulation of coumaric acid, depletion of aromatic amino acids phenylalanine and tyrosine matched the accumulation of enzymes of phenylpropanoid pathway (Figures 3E, 4A).

### Role of Cytokinin-Responsive Proteins in Response to Abiotic Stressors

The list of cytokinin-responsive proteins and metabolites indicated a similarity between cytokinin and an abiotic stimuli. Next, a set of experiments was designed to explore this putative crosstalk, including the response to salinity, temperature and drought. The same stringent protein quantitation criteria were applied, resulting in the identification of 308 stress-responsive proteins (Supplementary Table S1). Notably, the overlap between abiotic stress and cytokinin response was high, and only 76 cytokinin-responsive proteins were not considered to be differentially abundant in response to any of the abiotic stimuli (Supplementary Figure S1).

#### Salinity and Cytokinin Share Similar Response in Barley Root Proteome

The functional analysis (Figure 3D) indicated that 17 barley cytokinin-responsive proteins could be induced by salinity stress. In order to validate this potential crosstalk, seedlings were put for 24h into medium supplemented with 80 mM NaCl to elicit a salinity stress. The consecutive proteomics analyses revealed a statistical significant separation of salinity- and cytokinin-treated roots (Figure 4C), and a depletion and accumulation of 55 and 59 root proteins, respectively (Supplementary Table S1). The accumulated proteins included multiple glutathione S-transferases, glutathione reductase and superoxide dismutase, indicating an increase in the ROS metabolism. Roots under salinity stress also accumulated enzymes of phenylpropanoid biosynthetic pathway (phenylalanine ammonia-lyase, HORVU0Hr1G016330; alcohol dehydrogenase, HORVU6Hr1G035420), enzymes of energy metabolism, sulfur metabolism, and an ortholog of lipocalin that confers resistance to high salt in poplar (Abo-Ogiala et al., 2014). A decrease was found for proteins associated with cell wall organization or biogenesis, including several peroxidases or chitinase. In total, only four out of 17 putative salinity-responsive proteins were significantly changed under salinity stress, but the comparison with the response to cytokinin showed 26 similar changes, including an accumulation of a jasmonic acid biosynthetic enzyme (HORVU2Hr1G077220), phenylpropanoid biosynthetic enzymes, potassium channel subunit and a putative regulator of cell elongation (HORVU1Hr1G045630). A contrasting response was found for 15 proteins, including nuclease S1 and acid phosphatase (HORVU2Hr1G112830, HORVU4Hr1G085050; positive response to cytokinin).

#### Cytokinin Treatment Elicited Drought-stress Response in Barley Root Proteome

The cytokinin treatment resulted in a decrease of TCTP protein (HORVU4Hr1G033200) with a putative role in drought tolerance (Kim et al., 2012), and an increase in an osmoprotective compound mannitol and at least five proteins associated with the drought stress response. Next, in a parallel experiment, the three-day-old seedlings were deprived of water for 24 h, reaching the relative water content 83.2 ± 0.4% of control roots, and the identified cytokinin-responsive proteins were quantified (Tables 1, 2, Supplementary Table S1). The results confirmed that cytokinin-responsive proteins significantly overlapped with the drought-responsive subset which is well in line with the previous reports suggesting that cytokinin may induce osmotic stress hypersensitivity (Karunadasa et al., 2020). In total, out of 70 identified drought-responsive proteins, 39 were cytokinin-responsive and the response to cytokinin and drought was similar for 35 of these, including an accumulation of the subunit of a voltage-gated potassium channel (HORVU6Hr1G091250), annexin (HORVU6Hr1G074440) and depletion of ferredoxin–nitrite reductase (HORVU6Hr1G080750) and multiple enzymes of carbohydrate and amino acid metabolism.

#### Temperature-stress Response in Barley Root Proteome

Cytokinin signaling is an integral part of temperature-stress response, including heat stress, cold stress and temperature acclimation (Jeon et al., 2010; Macková et al., 2013; Černý et al., 2014; Danilova et al., 2016; Skalák et al., 2016). Here, 16 cytokinin-responsive proteins were associated with a temperature-stress. To provide evidence for this putative link, three-day old seedlings in magenta boxes were put onto an ice-bath or heated block, and temperature in the growth medium was kept for 120 min at 4 ± 2 and 30 ± 1°C to emulate cold- and heat-stress, respectively. The root tissue was harvested after 22 h of recovery at 20°C. Proteomics analysis revealed 152 and 108 cold- and heat-stress responsive proteins, respectively. Surprisingly, only 27 proteins were found to be differentially abundant in the both datasets and only seven of these manifested a temperature-dependent pattern, namely ribosomal protein S28 (HORVU3Hr1G115820), glutathione S-transferase (HORVU5Hr1G103420), peptidyl-prolyl cis-trans isomerase (HORVU6Hr1G077340), small heat shock protein (HORVU4Hr1G063350), aldehyde dehydrogenase 12A1 (HORVU1Hr1G080320), annexin 7 (HORVU6Hr1G074440) and asparagine–tRNA ligase (HORVU1Hr1G021130). The comparison with cytokinin-responsive proteins revealed a modest overlap, with 54 and 42 differentially abundant proteins for plants after a period of cold- and heat-stress, respectively. In total, out of 178 cytokinin-responsive proteins, 81 were found in the set of temperature-stress-responsive proteins, and most shared a similar response between temperature-stress and cytokinin treatment (Tables 1, 2, Supplementary Table S1, Supplementary Figure S1).

#### Response to Abiotic Stress and Cytokinin - Similarities in Proteome and Metabolome Patterns

Metabolome profiling showed a surprising similarity between cytokinin treatment and abiotic stress (Figures 4A,C). Notable similarity was found for the depletion of oxaloacetic acid and amino acids, including serine and proline. Proline is a well-known osmoprotectant, and its content should gradually increase upon prolonged stress. However, it has been shown that the initial free proline content in barley roots is very low and at least 48 h of salinity stress are required for any positive changes to be manifested (Ueda et al., 2007). The most similar metabolome response to cytokinin of the abiotic stresses examined was found in samples that experienced a period of cold stress, sharing 23 of 29 differentially abundant metabolites. Not all of the observed changes in roots under abiotic stress were deemed statistically significant (compared to the mock-treated controls; p < 0.05), but analysis of principal components confirmed that all treatments were distinct from the control, but not indistinguishable from each other (Figure 4B). In contrast, the comparison of proteome profiles by independent component analysis (Figure 4C) showed statistically significant separation of cytokinin treatment, salinity stress (IC1), and cold stress (IC2) from each other and both drought and heat stress that were similar to the mock control treatment. In total, 46 proteins were found with a similar and statistically significant response in at least three different treatments. These proteins included an enzyme of ascorbate biosynthesis (HORVU5Hr1G079230; positive response to temperature stress and drought), Hsp70-Hsp90 organizing protein (HORVU2Hr1G092190; positive response to cytokinin, cold- and drought-stress), annexin (HORVU6Hr1G074440; positive response to cytokinin, heat, drought and salinity), phenyl ammonium lyase (HORVU0Hr1G016330; positive response to cytokinin, heat and salinity) and a subunit of potassium channel (HORVU6Hr1G091250; positive response to cytokinin, drought, salinity). Only three proteins were found with a similar and statistically significant response in all experiments indicating that they have a role in the general response to external stimuli, namely thioredoxin-like protein (HORVU7Hr1G046280; depleted), NAD(P)H dehydrogenase (HORVU3Hr1G077250; depleted) and unknown protein HORVU2Hr1G098860 (depleted).

#### Cytokinin Treatment Reduced Hydrogen Peroxide Content in Roots

**FIGURE 5:**
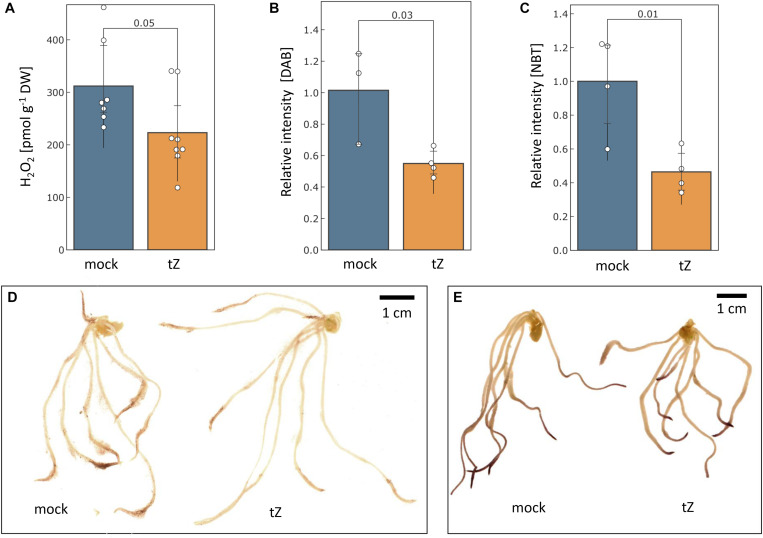
Cytokinin impact on the ROS production. The estimated mean content of hydrogen peroxide in barley roots determined by **(A)** PeroxiDetect Kit and **(B)** 3,3′-diaminobenzidine (DAB) staining; **(C)** Cytokinin impact on superoxide radical production as estimated by histochemical staining with nitroblue tetrazolium (NBT); Representative images of cytokinin- and mock-treated roots stained with DAB **(D)** and NBT **(E)**. Presented data are means and standard deviation of at least three biological replicates; Statistically significant differences (Student’s *t*-test) are indicated.

The measurement of aqueous hydrogen peroxide showed that the cytokinin treatment significantly reduced hydrogen peroxide content in barley roots by more than 25% (Figure 5A). The histochemical staining indicated that the reduction in the root tips could be even higher and a similar response was found for superoxide radicals (Figures 5B–E).

## Discussion

### Cytokinin Modulates ROS Metabolism in Barley Root

Changes in ROS metabolism enzymes have been previously reported in response to all major phytohormones (Černý et al., 2016), and it has been demonstrated that the ROS metabolism does not only represent a general stress response, but that it is required for mediating growth. For instance, phytohormone auxin promotes ROS production to facilitate cell wall loosening and cell elongation (Huan et al., 2016; Voxeur and Höfte, 2016). Here, out of more than 90 proteins involved in ROS metabolism, 11 were significantly differentially abundant in response to cytokinin, indicating an increase in the ROS production. Cytokinin application induced accumulation of all detected catalase isoforms (HORVU6Hr1G008640, HORVU7Hr1G121700), peroxidases (HORVU2Hr1G124970, HORVU1Hr1G016660), an enzyme that predicted to act on lipid peroxide-derived reactive aldehydes (HORVU3Hr1G015640), and a minor isoform of glutathione S-transferase (HORVU4Hr1G081100; less than 2% of the amount of all detected isoforms). Peroxiredoxin (HORVU3Hr1G037720) together with glutaredoxin (HORVU3Hr1G023970) and thioredoxin (HORVU7Hr1G046280) were depleted, as well as two peroxidases (HORVU2Hr1G125200, HORVU3Hr1G112350). The induction of ROS catabolism resulted in a decrease of hydrogen peroxide and superoxide radicals in the roots (Figure 5). This induction was likely triggered by a transient increase in the ROS production, which could correlate with an increase in spermidine and a depletion of its precursor putrescine (Figure 4A). Spermidine is a precursor of spermine (no difference in response to cytokinin detected) and both these polyamines reportedly mediate protection against oxidative damage caused by hydrogen peroxide (Rider et al., 2007). Polyamine production is also reportedly linked with an increase activity of catalase and glutathione S-transferase (Seifi and Shelp, 2019) which could coincide with the observed changes at the proteome level. Metabolomic data also indicate a higher turnover rate for glutathione, with a decrease of its precursor O-acetyl-serine and an increase in pyroglutamic acid (glutathione degradation product). However, a decrease in O-acetyl-serine could also be the result of cytokinin inactivation by zeatin 9-aminocarboxyethyltransferase which converts O-acetyl-serine and trans-zeatin into lupinic acid (Entsch et al., 1983). The existence of this enzyme found in Fabaceae has not yet been confirmed in barley, but the available data cannot exclude this possibility.

### Salinity and Drought Stress Elicit Cytokinin-like Response in Barley Root Proteome

Drought and salinity are globally the most frequent abiotic stresses, and both significantly impair crop yields. Barley is more resilient to salinity than other cereals with some cultivars tolerating up to 250 mM NaCl (Hanin et al., 2016). Here, we employed 80 mM NaCl that showed only a mild effect on the leaf area (less than 10% reduction at a prolonged treatment; results of preliminary experiments, data not shown) but still elicited a more potent response on the seedling root proteome than the water deprivation (Supplementary Table S1 and Supplementary Figure S1). Modulations of cytokinin metabolism or signaling can improve drought and salt tolerance, and it has been demonstrated that both the depletion of cytokinin and an increase in the cytokinin pool can promote plant growth under abiotic stress (e.g., Nishiyama et al., 2012; Prerostova et al., 2018). The exact mechanisms are not yet fully understood, but at least a part of the cytokinin promoted stress alleviation could reflect the priming of antioxidant systems (Nakabayashi et al., 2014). The similar priming was found here (as discussed above). Further, abundances of 48 cytokinin-responsive root proteins were found with a similar response under salinity stress or water deprivation (Supplementary Figure S1). It is thus tempting to speculate that this cytokinin-induced priming could be responsible for an enhanced resilience found in plants with modulated cytokinin pool.

### Cytokinin Has a Variable Role in Temperature Stress Response in Barley Root Proteome

The proteome profiling revealed that ca. 5% of the root proteome is formed by heat shock proteins. Heat shock proteins are ubiquitous and widely spread proteins across all taxonomic kingdoms. These proteins were first discovered in the response to an increase in temperature, but accumulated evidence indicates that they are involved in diverse processes. Besides their chaperon functions, HSPs participate in proteasomal degradation, protein-protein interactions and may also play a role in signaling cascades (e.g., McLoughlin et al., 2019; ul Haq et al., 2019; Tichá et al., 2020). Here, seven heat shock proteins (representing ca. 10% of the total identified HSP protein amount) were significantly depleted in response to cytokinin, including three and four representatives of the HSP70 and sHSP family, respectively. Proteins HSP70-6 (an ortholog of HORVU4Hr1G089090 and HORVU4Hr1G012460) and HSP21 (HORVU4Hr1G063350) were found to be essential for chloroplast development and thermotolerance in germinating seeds of Arabidopsis (Latijnhouwers et al., 2010; Zhong et al., 2013). In general, the exogenous cytokinin treatment elicited (at least partially) a low-temperature response in barley roots, which was manifested in the observed significant changes at the metabolome level and 37 proteins with a cytokinin-like response under cold stress. However, all four cytokinin-responsive sHSP in barley roots were accumulated after a period of cold-stress and not affected or depleted in response to a period of heat. This indicates a variable role of cytokinin in temperature stress response and is well in line with the previous contrasting reports of cytokinin role in cold- and heat-shock response and temperature acclimation.

### Cytokinin May Act as a Switch in the Phenylpropanoid Pathway

Cytokinin suppressed membrane steroid binding protein 1 (HORVU1Hr1G045630), an ortholog of Arabidopsis MSBP1 which regulates lignin biosynthesis, negatively regulates brassinosteroid signaling and cell elongation (Yang et al., 2005; Gou et al., 2018). A downregulation of MSBP in *Arabidopsis* resulted in a lower lignin deposition, and the accumulation of soluble phenolics in the monolignol branch (Gou et al., 2018). This would explain the observed significant accumulation of 4-coumaric acid which was specific for cytokinin treatment (Figure 4A). It has been previously reported that cytokinin signaling could result in a reduced lignification (reviewed in Didi et al., 2015), and results reported here indicate that MSBP could be its direct target. There are at least two plausible explanations for this cytokinin effect supported in the barley root dataset. First, lowering lignification could coincide with the cytokinin-induced accumulation of expansin B2 (HORVU1Hr1G054240) which is expected (by similarity) to cause loosening and extension of plant cell walls. Alternatively (or in parallel), cytokinin-induced reduction of lignin biosynthesis could be a switch in the phenylpropanoid pathway, promoting anthocyanin production, which is a well-known cytokinin response (Deikman and Hammer, 1995). This alternative is supported by the accumulation of phenylpropanoid biosynthetic enzymes, including two isoforms of phenylalanine ammonia-lyase 1 (HORVU0Hr1G016330, HORVU6Hr1G058820) or flavone 3′-O-methyltransferase 1 (HORVU7Hr1G082280).

## Conclusion

The cytokinin response has been extensively characterized in the model plant *Arabidopsis thaliana*, but an equivalent study of a crop plant has been largely missing. This work provided the first insight into the cytokinin-responsive proteins and metabolites in developing roots of barley seedling. The observed overlap with known cytokinin-responsive genes and proteins showed that this could be an excellent model for identifying hormone-responsive proteins and for the analysis of intensive crosstalk between plant hormones and abiotic signaling pathways.

## Materials and Methods

### Plant Material

Grain samples of *Hordeum vulgare* L. sensu lato variety (Sebastian) were obtained from field grown plants from the breeding station Stupice (Czechia) in 2016 and were stored in a sealed container at 4°C. For germination assay, grains were imbibed in 4 ml water supplemented with 5 × 10^–4^% (v/v) dimethyl sulfoxide (mock buffer) or 1 μM phytohormone (tZ - trans-zeatin; IAA - indole-3-acetic acid; GA3 - gibberellic acid; ABA – abscisic acid; Duchefa) in dimethyl sulfoxide (final concentration, as for the mock) and incubated at 20°C for 48 h in dark. For root proteome and metabolome analysis, grains were surface-sterilized (2% hypochlorite), imbibed and stratified at 4°C for 48 h. Stratified seeds were transferred onto half-strength Murashige & Skoog medium and placed in a growth chamber providing 20°C and 16/8 h light/dark cycles with 100 μmol m^–2^ s^–1^ photon flux density during light periods. After 72 h, sets of 10 germinated seedlings were exposed to 1 μM trans-zeatin or an abiotic stress by exposure to: 30°C or 4°C temperature for 2 h followed by a 22 recovery period at 20°C; medium supplemented with 80 mM NaCl (final concentration); or drought (by transfer to a dry Magenta box). 24 h after each treatment, the root tissue was dissected from the shoot, frozen and stored at −80°C. All experiments were carried out in at least three biological replicates, each consisting of 10 seedlings per sample.

### Protein Extraction and LC-MS Proteome Profiling

Total protein extracts were prepared as previously described (Hloušková et al., 2019) employing a combination of phenol/acetone/TCA extraction. Portions of samples corresponding to 5 μg of peptide were analyzed by nanoflow reverse-phase liquid chromatography-mass spectrometry using a 15 cm C18 Zorbax column (Agilent), a Dionex Ultimate 3000 RSLC nano-UPLC system (Thermo) and a qTOF maXis Impact mass spectrometer (Bruker) as previously described (Dufková et al., 2019). Peptides were eluted with up to a 120-min, 4% to 40% acetonitrile gradient. MS spectra were acquired at 2 Hz, while MS/MS spectra were acquired between 10–20 Hz using an intensity-dependent mode with a total cycle time of 7 s. The acquired spectra were recalibrated and searched against the reference barley (Mascher et al., 2017) by Proteome Discoverer 2.0, employing Sequest HT with the following parameters: Enzyme - trypsin, max two missed cleavage sites; Modifications - up to three dynamic modifications including Met oxidation, Asn/Gln deamidation, N-terminal acetylation, Met-loss (protein N-terminus), Met-loss + Acetylation (protein N-terminus); MS1 tolerance −35 ppm, MS2 tolerance −0.1 Da (Sequest). The quantitative differences were determined by the spectral counting method, followed by normalization and t-test (compared to the mock-treated roots; *p*-value < 0.05). For selected candidate proteins, the corresponding peptide peak areas were evaluated in Skyline (Pino et al., 2020). The proteomic data acquired have been deposited to the ProteomeXchange Consortium^1^ via the PRIDE partner repository (Vizcaíno et al., 2016) with the dataset identifier PXD020627.

### Metabolite Extraction and Analysis

Polar metabolites were extracted as previously described with few modifications (Cerna et al., 2017) and measured using a Q Exactive GC Orbitrap GC-tandem mass spectrometer and Trace 1300 Gas chromatograph (Thermo Fisher). Samples were injected using the split mode (inlet temperature 250°C, splitless time 0.8 min, purge flow 5.0 ml/min, split flow 6.0 ml/min) onto TG-5SILMS GC Column (Thermo Fisher, 30 m × 0.25 mm × 0.25 μm) with helium as a carrier gas at a constant flow of 1.2 ml/min. Metabolites were separated with a 28 min gradient (70°C for 5 min followed by 9°C per min gradient to 320°C and finally 10 min hold time) and ionized using the electron ionization mode (electron energy 70 eV, emission current 50 μA, transfer line and ion source temperature 250°C). The MS operated in the full scan mode, 60000 resolution, scan range 50–750 m/z, automatic maximum allowed injection time with automatic gain control set to 1e6, and lock mass [m/z]: 207.0323. Data were analyzed by TraceFinder 4.1 with Deconvolution Plugin 1.4 (Thermo) and searched against NIST2014, GC-Orbitrap Metabolomics library and inhouse library. Only metabolites fulfilling identification criteria (score ≥ 75 and ΔRI < 2%) were included in the final list.

### Determination of Hydrogen Peroxide and ROS Production

The lyophilized root tissue was homogenized and aliquots corresponding to 30–40 mg were analyzed using PeroxiDetect Kit (Sigma-Aldrich) according to the manufacturer’s instructions. The distribution of hydrogen peroxide and superoxide radical was visualized by endogenous peroxidase-dependent histochemical staining using 3,3′-diaminobenzidine (e.g., Novák et al., 2013) and nitroblue tetrazolium, respectively (Zhang et al., 2014). The staining intensity was quantified using ImageJ 1.53e (Schneider et al., 2012).

### Statistical Analyses

The reported statistical tests were generated and implemented using Instant Clue (Nolte et al., 2018), Rapid Miner (www.rapidminer.com; Mierswa et al., 2006) and Proteome Discoverer. Significant differences refer to *p* < 0.05.

## Data Availability Statement

The datasets presented in this study can be found in online repositories. The names of the repository/repositories and accession number(s) can be found below: https://www.ebi.ac.uk/pride/archive/, PXD020627.

## Author Contributions

MC and MB designed research. MC, MB, and HD performed research. MC, MB, ML, JN, IS-F, AR, and BB analyzed data. MC prepared figures and wrote the manuscript. All authors contributed to the article and approved the submitted version.

## Conflict of Interest

The authors declare that the research was conducted in the absence of any commercial or financial relationships that could be construed as a potential conflict of interest.

## Footnotes

1 http://proteomecentral.proteomexchange.org

## Supplementary Material

Supplementary Figure 1 Differentially abundant proteins in response to cytokinin or abiotic stimuli. Venn diagram summarizing **(A)** all identified differentially abundant proteins; **(B)** significantly accumulated proteins; and **(C)** significantly depleted proteins.

Supplementary Table 1 Differentially abundant proteins in response to cytokinin or abiotic stimuli. Supplementary table to Figures 1, 3, 4 and Tables 1, 2.

Supplementary Table 2 Results of proteome profiling. All identified proteins and peptides. Supplementary table to Figure 1 and Figure 3.

Supplementary Table 3 Results of metabolome profiling. Supplementary table to Figure 4.
