# Supplementary figures and images for: Barley Root Proteome and Metabolome in Response to Cytokinin and Abiotic Stimuli
Source: Front Plant Sci. 2020 Oct 28;11:590337. doi: 10.3389/fpls.2020.590337 (PMC7673457; doi:10.3389/fpls.2020.590337)

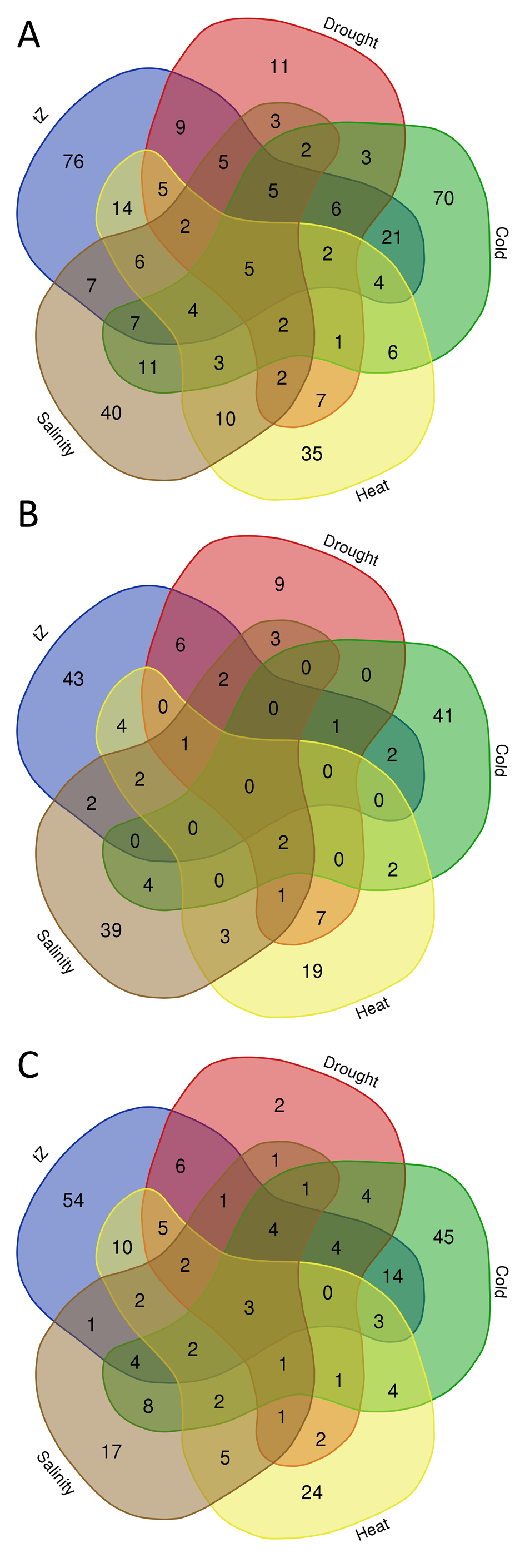

Supplement: Supplementary Figure 1 — Differentially abundant proteins in response to cytokinin or abiotic stimuli. Venn diagram summarizing (A) all identified differentially abundant proteins; (B) significantly accumulated proteins; and (C) significantly depleted proteins. [file Image_1.TIF]
